# NoC simulation steered by NEST: McAERsim and a Noxim patch

**DOI:** 10.3389/fnins.2024.1371103

**Published:** 2024-06-20

**Authors:** Markus Robens, Robert Kleijnen, Michael Schiek, Stefan van Waasen

**Affiliations:** ^1^Central Institute of Engineering, Electronics and Analytics: Electronic Systems (ZEA-2), Forschungszentrum Jülich GmbH, Jülich, Germany; ^2^Peter Grünberg Institute: Neuromorphic Compute Nodes (PGI-14), Forschungszentrum Jülich GmbH, Jülich, Germany; ^3^Faculty of Engineering, Communication Systems, Duisburg-Essen University, Duisburg, Germany

**Keywords:** neuromorphic computing, network-on-chip simulator, multicast, co-simulation, latency, spiking neural networks

## Abstract

**Introduction:**

Great knowledge was gained about the computational substrate of the brain, but the way in which components and entities interact to perform information processing still remains a secret. Complex and large-scale network models have been developed to unveil processes at the ensemble level taking place over a large range of timescales. They challenge any kind of simulation platform, so that efficient implementations need to be developed that gain from focusing on a set of relevant models. With increasing network sizes imposed by these models, low latency inter-node communication becomes a critical aspect. This situation is even accentuated, if slow processes like learning should be covered, that require faster than real-time simulation.

**Methods:**

Therefore, this article presents two simulation frameworks, in which network-on-chip simulators are interfaced with the neuroscientific development environment NEST. This combination yields network traffic that is directly defined by the relevant neural network models and used to steer the network-on-chip simulations. As one of the outcomes, instructive statistics on network latencies are obtained. Since time stamps of different granularity are used by the simulators, a conversion is required that can be exploited to emulate an intended acceleration factor.

**Results:**

By application of the frameworks to scaled versions of the cortical microcircuit model—selected because of its unique properties as well as challenging demands—performance curves, latency, and traffic distributions could be determined.

**Discussion:**

The distinct characteristic of the second framework is its tree-based source-address driven multicast support, which, in connection with the torus topology, always led to the best results. Although currently biased by some inherent assumptions of the network-on-chip simulators, the results suit well to those of previous work dealing with node internals and suggesting accelerated simulations to be in reach.

## 1 Introduction

“Communication is the key”—this paradigm is quite ubiquitous nowadays. It does particularly apply to the wealth of neuro-inspired computing applications, which receive a lot of attention, currently. All of them are characterized by the use of decentralized computing resources and memory. While this concept will challenge communication facilities of the system in general, the situation is exacerbated by the high number of synaptic connections in large-scale neuromorphic computing platforms dedicated to theoretical neuroscience. Prior to design of the current generation of these systems, there have thus been studies to assess the capabilities of their communication networks—often using particular specification languages. In Merolla et al. (2014), for example, the multicast binary-tree network used in Neurogrid is discussed and analyzed. For its design, a synthesis method based on the high-level language Communicating Hardware Processes (CHP) was applied. Rationales behind the communication architecture used in BrainScaleS—featuring a combination of spatial and temporal multiplexing—are presented and analyzed in Schemmel et al. (2008) as well as in Fieres et al. (2008). Details on the communication system of SpiNNaker are provided in Furber et al. (2006) and in Plana et al. (2008), for example. Conceptually, it is based on an associative multicast router, the benefits of which are evaluated in Navaridas et al. (2012) using an analytical approach, and in Lester and Richards (2008) using a formal specification written in Concurrent Haskell. In Moradi et al. (2018), the connectivity scheme of a DYNAPs architecture is backed on analytical considerations regarding memory requirements, while the components of the hierarchical routing network are synthesized following the Communicating Hardware Processes (CHP) formalism. As a last example, the online computation of synaptic connectivity using (multidimensional) table-based pseudo-random number generators within the IBM INC-3000 system is proposed in Heittmann et al. (2022) and examined by a quantitative design-space exploration based on a high-level synthesis (HDL) logic design methodology.

These approaches did a great job in optimizing the communication facilities of the physical implementation in many aspects. For the investigation of large-scale neural networks, however, it is often desirable to run simulations as fast as possible, e.g., to explore slow processes like structural plasticity and long-term memory. In this study, we therefore put our focus on further reductions in latencies. In this context, and with a certain set of desired neuroscientific test cases in mind, trading flexibility against improved latencies has thus been deemed an interesting option. This adds another dimension to the initial paradigm, since it calls for excellent communication between the different research disciplines involved. Within the realm of computational neuroscience, software tools with well-defined interfaces have been developed to discover the interaction of neurons, synapses, and neural networks. Such tools are ideal to specify and execute the desired test cases to generate realistic traffic patterns that need to be transferred over the connections of the envisaged system. At the same time, a couple of network-on-chip (NoC) simulation platforms exist that can provide statistical information on such transfers and point to potential bottlenecks in possible hardware implementations. With the reduction of communication latencies in mind, it is now highly desirable to link these simulation frameworks in such a way that they provide useful insights even for non-domain experts.

Inspired by Lahdhiri et al. (2020) and Balaji et al. (2020), we therefore created McAERsim as well as a patched version of the NoC simulator Noxim (Catania et al., 2015) that accept spike traces generated by the generic neuroscientific development environment NEST (Gewaltig and Diesmann, 2007). In Lahdhiri et al. (2020), a framework combining the system level simulator Sniper (Carlson et al., 2011) with the NoC simulator Noxim is proposed to collect statistics regarding network traffic as well as energy consumption caused by application defined communication patterns. However, the released version of Noxim expects a traffic table for this purpose, which solely contains a packet injection rate for each source / destination pair. This way, only first order statistics of the individual communications are preserved. As opposed to that, *Noxim++*, embedded in the design methodology SpiNeMap (Balaji et al., 2020), is capable of communicating spike packets created according to the output traces of CARLsim (Chou et al., 2018), another application-level simulator for large-scale, biologically detailed spiking neural networks. To allow for such communication and to incorporate additional features, Noxim is extended within this framework and consequently called *Noxim++*. Its core purpose within SpiNeMap is to provide energy and latency estimates for different mappings of neuron clusters to hardware (SpiNePlacer) that were algorithmically defined to reduce the number of spikes on the global interconnect (SpiNeCluster). The computed key figures are used as fitness function in an instance of the particle swarm optimization (PSO; Eberhart and Kennedy, 1995). Since inter spike interval distortion depends on latency, it is minimized as well by this approach. McAERsim and the Noxim patch were developed with similar goals like *Noxim++* but interfacing with NEST rather than with CARLsim by default.

Like CARLsim, NEST has a large user community—especially in the field of computational neuroscience. Thus, certain sets of neuroscientific test cases may be of special importance, as has been outlined above. To pose a low threshold in examining potential network traffic caused by them, we selected to connect NEST with the network-on-chip simulators. At the same time, the network-on-chip simulators are based on Noxim, because of its versatile parameter options as well as its comparably simple and well-organized code. The first aspect has been highlighted by a number of NoC simulator comparisons, for example, in Gu (2011); Huynh (2016); Khan et al. (2018); Lahdhiri et al. (2020); Lis et al. (2011), while the second aspect has been stressed in Huynh (2016). A simulation based comparison of different network simulators in addition can be found in Khan et al. (2018).

While the Noxim patch basically is intended to enable the required interfacing,^1^ McAERsim leans on the infrastructural and organizational concepts of Noxim but may be considered a full re-write to cover tree-based hardware multicast support for packets encoded according to the address event representation (AER; Mahowald, 1992). Multicast (Navaridas et al., 2012; Merolla et al., 2014) as well as mixtures of unicast and broadcast routing (Moradi et al., 2018; Heittmann et al., 2022) are preferred packet forwarding schemes in large-scale neuroscientific simulation platforms. They are of increasing importance for certain workloads in conventional NoCs as well (Jerger et al., 2008; Krishna et al., 2011), but—according to the same publications—most improvements in NoC architectures only have been devoted to unicast traffic. In addition to more complex routing decisions that need to account for possible switching-level deadlocks (Konstantinou et al., 2021), for example, this may be the reason, why multicast support in NoC simulators is limited. Accordingly, in (Agarwal et al., 2009, Section 2.1), it is stated that hardware multicast support—as research in progress—is not modeled inside the routers of Garnet. At the time of writing, this simulator is shipped as one of two build-in options for the interconnection network model within the Ruby Memory System of gem5, a platform for computer-architectures research. The simple network model, which is the second and default option, assumes perfect hardware multicast support (Martin et al., 2005; Agarwal et al., 2009) but abstracts out detailed modeling of the switches' microarchitecture. There is a third simulator, Topaz, that can be interfaced with gem5 via the Ruby Memory System. According to (Abad et al., 2012, Table I), it does provide tree-based multicast support. However, its documentation (Abad, 2022) reveals that this capability relies on routing masks, which confine possible network sizes to 8 × 8 nodes. If no hardware multicast support is available, respective packets usually get broken into multiple unicast messages at the network interface (Jerger et al., 2008; Lowe-Power, 2022) leading to performance penalties. These examples show that there was a need for a network-on-chip simulator like McAERsim with hardware multicast support and a detailed microarchitectural router model as reflected by the router pipeline model discussed in later sections of this study. In conjunction with the Noxim patch, it allows for a convenient design space exploration, using application defined traffic as well as different network topologies and casting schemes.

By extrapolation from the results we obtained with this approach for the cortical microcircuit model (Potjans and Diesmann, 2014) scaled to 33%, we assume that it can be used for biological networks up to the scale of the full cortical microcircuit. According to Heittmann et al. (2022), the cortical microcircuit may be considered a unit cell of the cortical network in that it reveals a distinct local structure with a significant fraction of local synapses and an associated biological volume that is representative for the ranges of local synapses established by a neuron. This work may thus be considered a supplement to some recent achievements reported earlier. In Kleijnen et al. (2022), we presented a Python-based network simulator for large-scale heterogeneous neural networks and applied it to the evaluation of network traffic caused by the multiarea model (Schmidt et al., 2018). This model is composed of 32 areas, which—loosely speaking—may be considered special instances of the cortical microcircuit with some extra connectivity. Latency is reported in terms hops by the Python-based simulator, which is a reasonable estimate for the spike delivery times as long as congestion does not occur. Therefore, we conceived a hierarchical approach, in which possible hot spots or bottlenecks are identified by our Python-based simulator. Further analysis by cycle-accurate simulations is then possible with the proposed framework. In addition, this work relates to Trensch and Morrison (2022), which discusses conception, implementation, and performance modeling of a so called “Hybrid Neuromorphic Compute (HNC)” node. Specifically, Trensch and Morrison (2022) focuses on efficient intra-node spike delivery incorporating DRAM memory that is external to the programmed system-on-chip (SoC) but forms a node together with it. Supported by latency measurements, a performance model for the HNC is defined and extended to mimic cluster operation. This extension assumes a fixed inter-node transmission latency *T*_*COM*_ but adds a small fraction α·*T*_*COM*_ per spike event on top to account for a workload dependent latency increase. In Trensch and Morrison (2022), *T*_*COM*_ is set to 500ns, assuming a simulated time step of 0.1ms, a simulation acceleration factor of 100, and an equal distribution of the resulting time interval between computation and communication tasks.

Applying the proposed framework, we will conduct some complementary studies with respect to spike delivery. We will evaluate to which extend inter-node communication can fulfill the premise of *T*_*COM*_ = 500ns. Lead by this aim, we will examine how inter-node communication latencies depend on parameters like network topology and casting type. Since these simulations have not been performed for the full scale cortical microcircuit so far, we will also observe how latencies vary due to different scaling factors and network partitionings. In addition, we will present how the network parameters can affect network loads and the homogeneity of their distribution.

The remainder of this article is organized as follows. Section 2 provides short outlines on the involved simulators and discusses the most important modifications that were necessary to embed them into the frameworks. Furthermore, a router pipeline model will be discussed in detail, which is at the heart of the tree-based source-address driven multicast router of the newly conceived simulator McAERsim. Prior to a discussion of the results obtained by the execution of the frameworks, hardware environments, batch processing, as well as parameter choices will be considered in Section 3. Finally, Section 4 will classify the results with respect to the aim of supporting the development of an accelerated large-scale neuroscientific simulation platform. It will also reveal some improvement potential encouraging some future work.

This study tries to enable the reader to easily reproduce our results and to use our approach for further research and development. The reader who is mainly interested in the results may concentrate on the introduction, the results section, and the discussion (last section).

## 2 Methods

To enable the desired co-simulations that have been outlined in the introduction, we tried to keep the necessary modifications to the source codes of the involved simulators as small as possible.^2^ However, since Noxim does not model tree-based hardware multicast support, we created McAERsim, a network-on-chip simulator with tree-based hardware multicast support for AER encoded packets and the capability to stimulate a simulation based on output files generated by NEST. An additional feature are multi-radix routers that allow multiple processing elements to be attached to a single router. The individual simulators as well as the modifications applied to them will be discussed in the following subsections in more detail.

### 2.1 NEST

**Figure 1:**
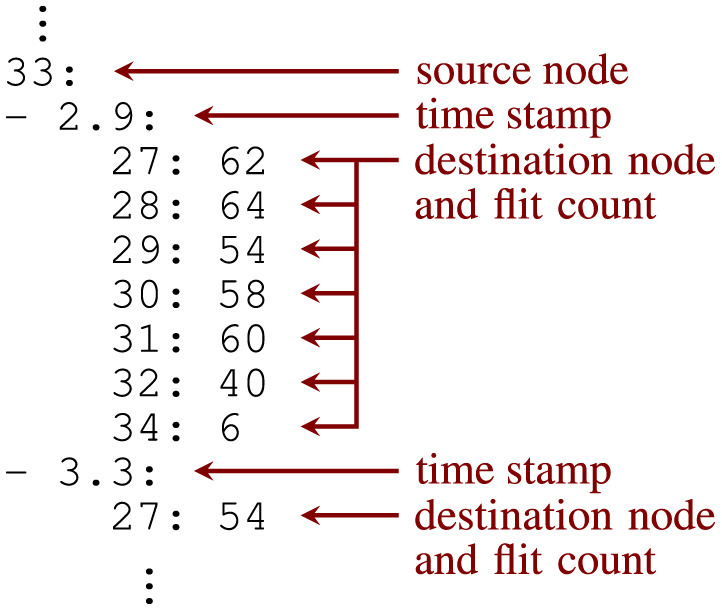
Excerpt from the YAML file generated by the modified helpers.py script of NEST's cortical microcircuit model intended for the Noxim based simulations.

The Neural Simulation Tool (NEST) is a simulator for large, heterogeneous spiking neural networks emulating an electrophysiological experiment and its outcome. For this purpose, NEST provides different neuron and synapse models, featuring spike-timing dependent plasticity as well as short-term facilitation and depression, for example. While the simulation kernel is written in C++, there are three user interfaces, accepting commands from different languages. Natively, NEST uses the simulation language interpreter (SLI). In addition, there is a front-end for the interpreted programming language Python (PyNEST), which is currently rather popular. Finally, there is also support for PyNN (Davison, 2008) targeting a simulator-independent description of the neural simulation with Python commands. Problems of current relevance to the computational neuroscience community are often specified in terms of test cases, a couple of which have been transferred into one of the languages that can be passed to NEST. A popular example is the cortical microcircuit model (Potjans and Diesmann, 2014), which is contained in the examples subdirectory of PyNEST. The execution of this model is split into phases, the last one being the evaluation phase. During this phase, auxiliary functions defined in the file helpers.py are applied to the simulation outputs for further processing. To generate the YAML file that is used for data exchange between PyNEST and the patched version of Noxim, intermediate results created by these auxiliary functions can be reused. Therefore, three additional functions have been defined and appended to this file. Figure 1 shows an excerpt from the file they generate and explains its simple file format.

Note that a list has been used at the second hierarchy level so that the chronological order of time stamps is preserved. It is also worth mentioning that only one of the python scripts defining the test case had to be modified, while the source files of the simulation kernel were left unchanged. In fact, two versions of this YAML file are generated. The first one is used for destination-address driven casting types: unicast (UC) and local multicast (LMC), while the second version is applied to the source-address driven casting type: local multicast (LMC_SRC). They differ in the number of flits to be transmitted, which is equal to the number of neurons in the target node that need to be reached from spiking neurons of the source node in the first case, and which is equal to the number of spiking neurons within the source node that have connections to the target node in the second case. McAERsim, on the other hand, uses a couple of NEST output files directly. In addition, it just requires a connectivity file. Keys at the top level of this YAML file represent NEST's neuron population IDs, while a key value pair in a nested dictionary associates a source neuron ID with all its destination neuron IDs. This connectivity file is created by an alternative helpers.py contained in a second copy of the examples directory.

### 2.2 Patched version of Noxim

Noxim is a network-on-chip (NoC) simulator based on SystemC that supports direct and indirect wired network topologies as well as wireless connections (Catania et al., 2015). The former consist of interconnected tiles, i.e., processing elements and/or routers, that are specified in plain SystemC, so that cycle-accurate simulations are possible. Wireless communications are enabled by the addition of hubs and channels that make use of the Transaction Level Model (TLM) library of SystemC and allow for the specification of transaction delays this way. As a simulation outcome, statistics on network traffic and energy consumption are collected and displayed.

**Figure 2:**
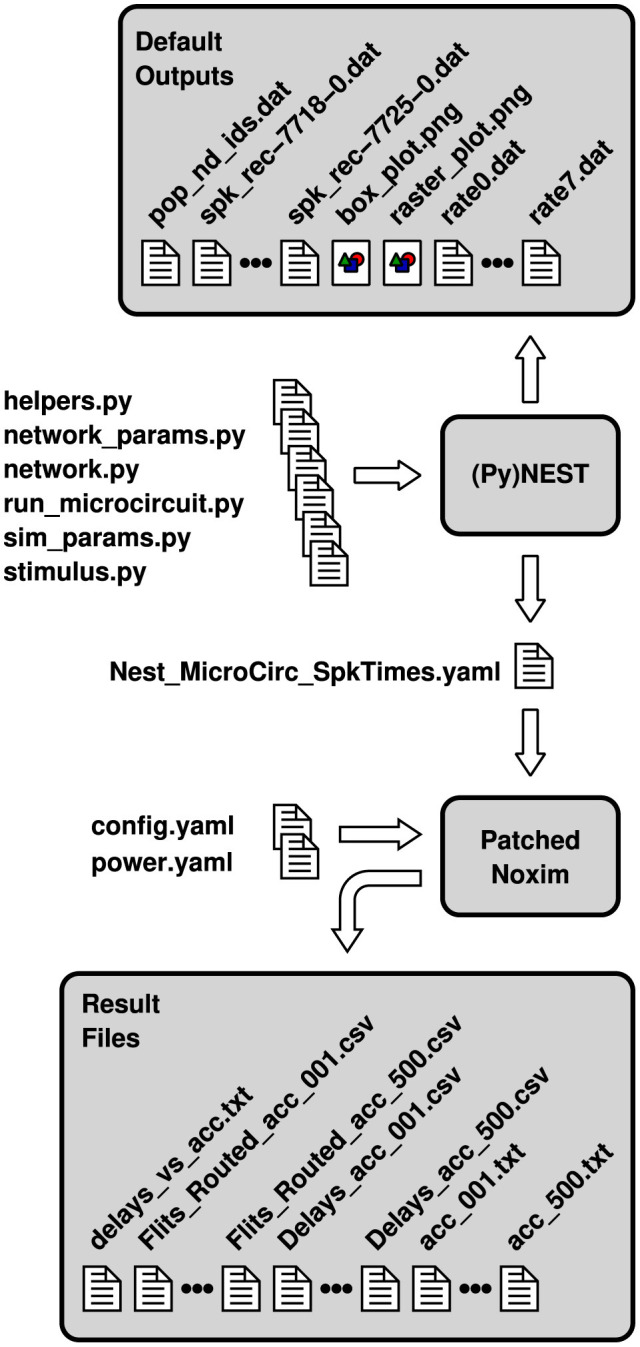
Configuration and result files involved in a co-simulation of NEST and a patched version of Noxim. File names are partially abbreviated—usually, they encode topology, size and the number of neurons per node as well.

The bulk of simulations is controlled by a single configuration file, config.yaml, while a second configuration file, power.yaml, contributes the parameters necessary for the energy estimates (cf. Figure 2). They are determined either based on measurements or based on extraction results from register transfer level (RTL) implementations. During the initial design space exploration, these parameters are usually unknown, so that there is less user interaction with power.yaml. By editing the configuration file config.yaml, however, or by passing equivalent command line arguments to Noxim, not only the network topology can be selected, but also different sizes, the number of virtual channels, a deterministic or adaptive routing scheme, a selection strategy, the traffic pattern, wireless network parameters, as well as further simulation parameters can be set.

In the released version of Noxim, trace-based traffic can only be specified by packet injection rates for source/destination pairs, i.e., by statistical means. In general, however, traffic is launched into the network by the processing elements—or, more precisely, from the canShot(Packet& packet)-method that is called from the SystemC method process txProcess() within ProcessingElement.cpp. During each clock cycle, a comparison to a random number is performed in this method, which decides on the packet emission. This makes it simple to add a case in which a packet is launched based on its time stamp rather than the outcome of this random experiment. For this purpose, an additional class called GlobalNestTrace has been created, the core attribute of which associates source node IDs with traffic queues.^3^ Supported by the format of the YAML file that is used for data exchange between NEST and Noxim (Figure 1), reading these data into memory is simple as well and results in a population-wise sequential mapping of neurons to the processing elements. Since time stamps of a NEST simulation are recorded in milliseconds, whereas they are specified in picoseconds in Noxim, an appropriate multiplier has to be used for the conversion during this step. This multiplier is stored in the global variable nest_time_multiplier and can be varied to mimic different acceleration factors.

Moreover, the destination-address driven casting types unicast (UC) and local multicast (LMC) as well as the source-address driven local multicast type (LMC_SRC) had to be introduced. As already discussed with respect to the YAML file in Section 2.1, they differ in their packet length and organization. In case of UC and LMC_SRC, single-flit packets are possible, raising the demand for an additional flit type, FLIT_TYPE_HEADTAIL, which has besides been implemented. The casting types take effect in the canShot method that is a member of the class GlobalNestTrace and that is called from the canShot method of a processing element in case of a simulation that is based on NEST time stamps.

Our results in Kleijnen et al. (2022) pointed to some advantages of torus shaped networks in comparison with standard mesh networks. Considering randomly connected neural networks, for example, the variability of network loads in torus shaped networks has been observed to be much less than in standard mesh networks. To exploit these characteristics and to foster the hierarchical simulation approach outlined in the introduction, the Noxim patch introduces support for torus shaped networks as well. While deadlocks can not occur in mesh networks using XY routing, it is well-known that this does not apply to torus shaped networks (Mirza-Aghatabar et al., 2007). In the latter case, at least two virtual channels are required for a systematic exclusion of deadlocks. However, the minimum number of virtual channels suffices only, if packets are allowed to change virtual channels during turns. In Noxim, usually a packet stays within the same virtual channel once it has been assigned to it. As a workaround, we thus use four virtual channels for the torus shaped network and assign one of them to a packet depending on the wrap-around connections it will take. In addition, the routing algorithm itself needs to be aware of the additional connections and make appropriate use of them, so that an adaptation of the XY routing algorithm had to be devised that is invoked by XY_TORUS in the file config.yaml or on the command line.

Finally, the aggregation of statistical data in Stats.h/.cpp and GlobalStats.h/.cpp has been extended to enable the creation of the plots shown in Section 3. Basically, this called for a transfer of all transmission delays (latencies) into a single vector. Furthermore, some means for data export had to be conceived, so that this vector as well as the matrix of routed flits can be exported as comma separated value (CSV) file.

The whole workflow for a co-simulation involving NEST and the patched version of Noxim is illustrated in Figure 2. The Python scripts that are fed into PyNEST are those of the pynest/examples/Potjans_2014 subdirectory of NEST, except for the modifications to helpers.py that have been discussed in Section 2.1 and some minor modifications to network.py as well as sim_params.py to call the respective functions. Steered by these inputs, PyNEST generates some data files as well as two portable network graphics (PNG) files by default. Due to the modifications, it creates the two YAML files adhering to the file format of Figure 1 in addition. Depending on the casting scheme that shall be applied, one of these files together with the configuration files config.yaml and power.yaml can be used to invoke Noxim, display a summary on overall network statistics, compose the vector of transmission delays, and export the latter together with the matrix of routed flits as CSV files. The multitude of files marked as “Result Files” in Figure 2 is the outcome of a Bash script that exploits the command line interface of Noxim to step the global parameter nest_time_multiplier through different values and parse the overall statistics by sed.

Choices to set the simulation parameters were rather limited in the course of this procedure. With respect to NEST—or more precisely, its cortical microcircuit model—they were first kept at their default settings. This results in the number of neurons and in the number of synapses per neuron being scaled down both to 10%, which is the recommended size for first experiments using a standard desktop PC. With regard to the patched version of Noxim, they were set with the premise of maintaining consistency between the simulations. Based on the preceding scaling considerations, the dimensions of the NoC are determined, once the number of neurons per computation node *NpN* is defined. According to the findings in Heittmann et al. (2022) and Trensch and Morrison (2022), *NpN* = 256 was deemed a reasonable number. The network size was thus fixed to 6 × 6 tiles. To use a common width and to be able to accommodate addresses for the full scale cortical microcircuit later on, the flit size has been set to 32 bit. Buffers uniformly were assigned a depth of 8. According to Seitanidis et al. (2014), this value at least needs to agree with the flow-control round-trip latency, i.e., it needs to exceed the pipeline stage count by 2. If this condition is not fulfilled, throughput degradation occurs. In Peh and Dally (2001), this phenomenon is explained by increased buffer idle times, which reduce the effective amount of buffering. The number of virtual channels is stipulated by the implementation of torus shaped networks. However, according to the simulation results presented in Dally (1992) for the 16-ary 2-cube, most of the throughput gain in this setup is realized with four virtual channels, which supports this selection. Wireless connections have not been considered, so that related parameters do not need to be modified.

### 2.3 Router pipelines

**Figure 3:**
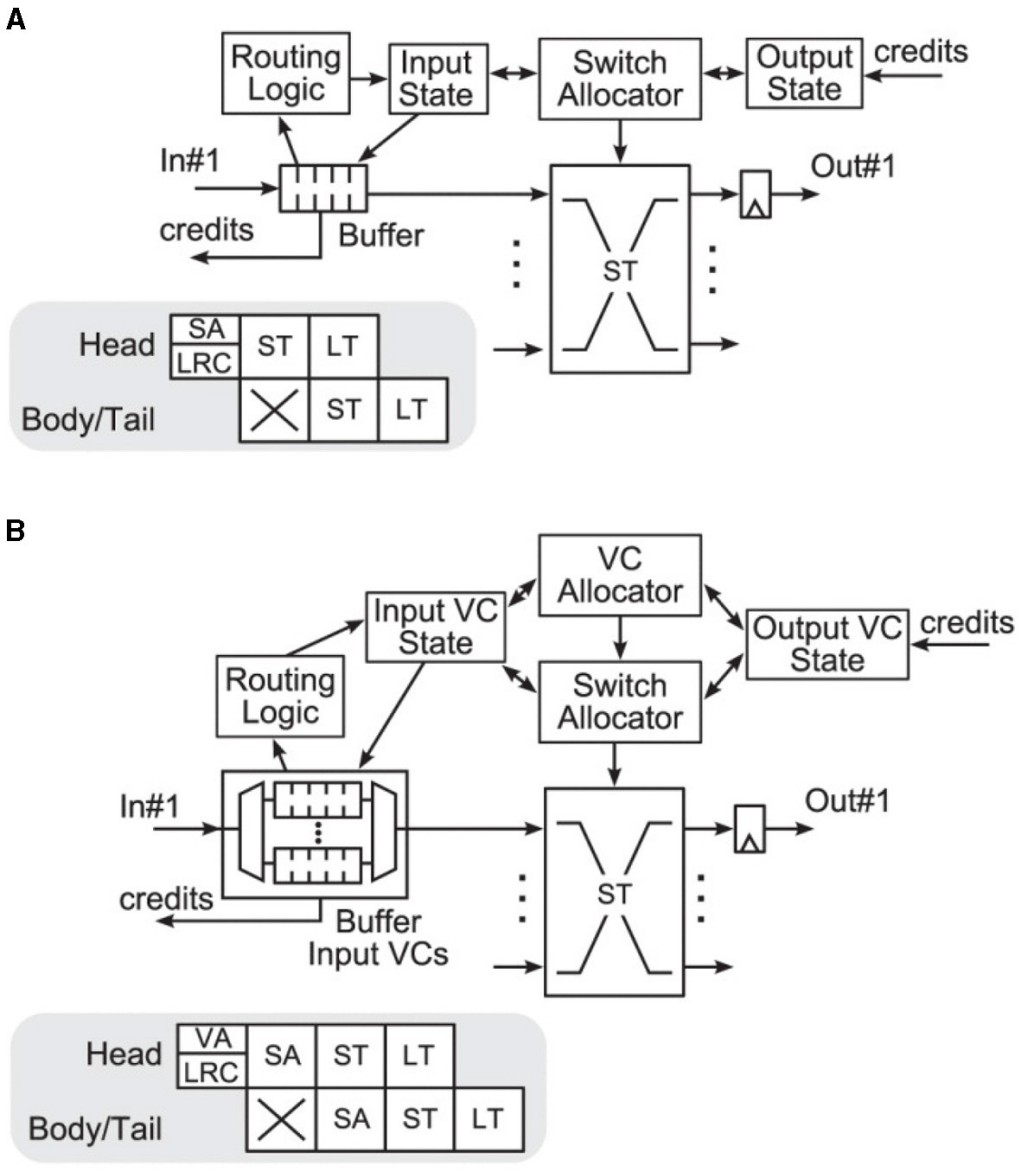
Canonical architectures according to Dimitrakopoulos et al. (2013); Peh and Dally (2001) of **(A)** a wormhole router and **(B)** a virtual channel router with separable allocation. The latter cycles through the following states: buffer write (BW), routing computation (RC), virtual channel allocation (VA), switch arbitration (SA), and switch traversal (ST). VA and SA are affected by credits reflecting the buffer status of the destination. The design of the router pipelines, shown in the gray boxes, assumes that destination decoding and routing computation are prepared in the previous router (lookahead routing computation—LRC). © 2013 IEEE. Reprinted, with permission, from Dimitrakopoulos, G., Kalligeros, E., and Galanopoulos, K. “Merged Switch Allocation and Traversal in Network-on-Chip Switches,” IEEE Transactions on Computers, Vol. 62, No. 10, pp. 2001–2012, Oct. 2013. doi: 10.1109/TC.2012.116.

The workflow presented in the previous subsection tries to meet the expectation of modest changes to the source codes of the involved simulators while it implements the required features. Consequently, the router model of Noxim is kept untouched. It adheres to the typical virtual channel router model with separable allocation outlined in Dimitrakopoulos et al. (2013); Peh and Dally (2001), for example, so that the header flit of a packet proceeds through all the states shown in Figure 3B, while body and tail flits inherit the routing information as well as the virtual channel from the header flit of a packet and skip through respective states.

**Figure 4:**
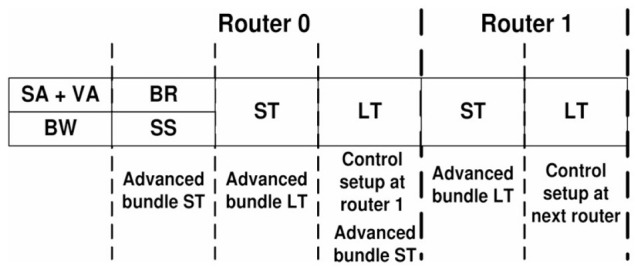
Single-cycle pipeline of Kumar et al. (2007). The states within this pipeline are buffer write (BW), switch arbitration and virtual channel allocation (SA+VA), buffer read (BR), switch setup (SS), switch traversal (ST), and line traversal (LT). The advanced bundle proceeds through some of these states one cycle earlier and performs a control setup at the next router. © 2007 IEEE. Reprinted, with permission, from Kumar, A., Kundu, P., Singh, A. P., Peh, L.-S., and Jha, N. K. “A 4.6Tbits/s 3.6GHz Single-cycle NoC Router with a Novel Switch Allocator in 65nm CMOS,” in IEEE Proceedings of the 2007 25th International Conference on Computer Design, Lake Tahoe, CA, USA, Oct. 7–10, 2007, pp. 63–70. doi: 10.1109/ICCD.2007.4601881.

Since routing computation (RT) can be performed in parallel to the buffer write (BW) operation, traditionally this leads to a five stage router pipeline (Agarwal et al., 2009, Figure 9). Following the simple analytical model of Seitanidis et al. (2014) or the parametric router delay equations of Peh and Dally (2001), the number of pipeline stages in this configuration is close to optimum with respect to latency. However, there have been numerous approaches to shorten this pipeline. In Kumar et al. (2007), it could be reduced to two stages using advanced bundles and pipeline bypassing, for example. Because the cycle spent during line traversal is usually not counted, it is referred to as single-cycle pipeline in the named publication, which is illustrated in Figure 4. Accordingly, it is stated in Lowe-Power (2022) that Garnet2.0 uses a state-of-the-art 1-cycle pipeline, in which buffer write (BW), routing computation (RT), switch arbitration (SA), virtual channel allocation (VA), as well as switch traversal (ST) all happen in one cycle, while line traversal (LT) takes place in the following cycle. Noxim follows a similar concept^4^ but implements BW in one cycle, while RC, SA, VA, ST, and LT all are performed in a second cycle.

**Figure 5:**
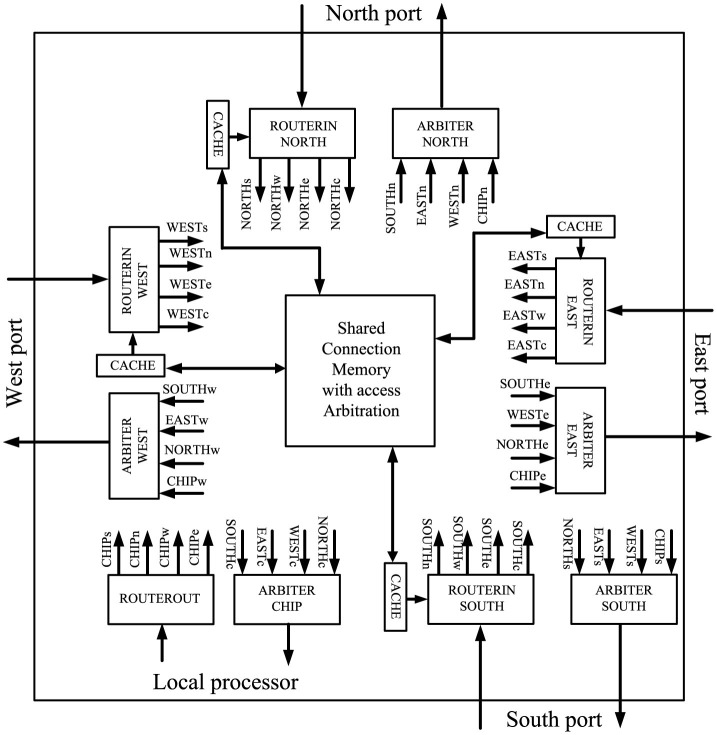
Source-address driven multicast router block diagram of Zamarreno-Ramos et al. (2013). Connections are labeled by source/destination pairs using the following abbreviations: w, west; n, north; e, east; s, south; c, core (local processing element). © 2012 IEEE. Reprinted, with permission, from Zamarreno-Ramos, C., Linares-Barranco, A., Serrano-Gotarredona, T., and Linares-Barranco, B. “Multicasting Mesh AER: A Scalable Assembly Approach for Reconfigurable Neuromorphic Structured AER Systems. Application to ConvNets,” IEEE Transactions on Biomedical Circuits and Systems, Vol. 7, No. 1, pp. 82–102, Feb. 2013. doi: 10.1109/TBCAS.2012.2195725.

**Figure 6:**
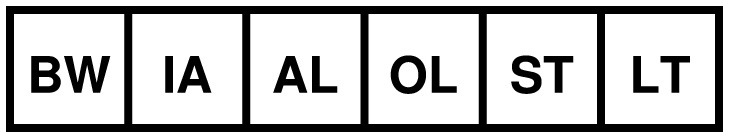
McAERsim's source-address driven tree-based multicast router pipeline consisting of five stages: Buffer write (BW), input arbitration (IA), address look-up (AL), (active) output look-up (OL), and switch traversal (ST). Finally, line traversal (LT) is performed.

Some assumptions that are inherent to the traditional five stage router pipeline of a conventional unicast router or improvements thereof, like in Figure 4, do no longer apply for source-address driven multicast routers designed for large-scale networks. The routing computation, for example, is usually performed on-the-fly in a unicast router, while it may be split over several cycles in a multicast router. McAERsim therefore uses a different pipeline model that has been derived from a simplified version of the source-address driven multicast router block diagram proposed in Zamarreno-Ramos et al. (2013), which is shown in Figure 5. The simplification is that no cache is used, which, in turn, allows for the substitution of the output arbiters by simple multiplexers composing a switch. A quote from (Heittmann et al., 2022, p. 8) underpins this decision: “in case of the microcircuit and a chosen micro-cluster size of 256 neurons, practically every incoming spike needs to be projected to at least one neuron in every micro-cluster.” This means that there is a large number of flows potentially leading to frequent cache misses. At the same time, the implied clustering does allow for a routing table minimization by taking advantage of the “don't care” states in ternary content addressable memory (TCAM) as discussed in Mundy et al. (2016), for example. Thus, a compact TCAM size is preserved and reasonable access times are ensured. While address lookups within one cycle are therefore presumed, the TCAM—as an implementation detail—is abstracted away from the source code of McAERsim. Rather, a C++ std::map is used. The address retrieved from the respective memory structure is then applied to access static random access memory (SRAM) as in Fritsch et al. (2015), which in turn contains a digital word with bit positions encoding activity of the different outputs as in Zamarreno-Ramos et al. (2013).^5^ If the combination of TCAM and SRAM forms a shared connection memory,^6^ only one input can be granted access to this memory per clock cycle, so that a need for arbitration between the inputs arises. Consequently, output activation as read from the shared memory always relates to a single input, which waives the demand for separate output allocation. The switch, or the multiplexers replacing the output arbiters in Figure 5 as one of its possible implementations, can then be traversed according to these settings. Finally, the AER events are sent along the lines that are connected to the active outputs. Assuming that the inputs are buffered, these considerations lead to the pipeline model depicted in Figure 6. In this model, a distinct pipeline stage is allotted to each of the components discussed before.

Cases in which input buffers of target nodes are unavailable prevent AER events from line traversal. A back-pressure mechanism similar to Plana et al. (2008) is then used to freeze most of the pipeline stages. However, the line traversal stage keeps trying to transmit the AER packet and re-enables the other stalled stages upon success.

### 2.4 McAERsim

McAERsim is a network-on-chip (NoC) simulator based on SystemC that provides source-address driven tree-based multicast support for AER packets in direct wired network topologies. It has been implemented in analogy to Noxim but focusses on AER packets, tree-based dimension ordered XY multicast routing, and network traffic that is externally generated, in particular by NEST. Just like Noxim, it features cycle-accurate simulations of networks consisting of interconnected tiles, i.e., processing elements and routers. However, unlike Noxim, it admits multiple processing elements per tile, which calls for higher-radix routers. In principle, it can provide similar statistics on network traffic and energy consumption as simulation output. Yet, lacking a detailed RTL implementation, the configuration file power.yaml currently is initialized to zero, so that our emphasis will be on network statistics.

The source-address driven multicast router model discussed in the second part of the previous subsection is at the core of McAERsim's simulation engine implementation, in which arbitration is done in a round robin fashion. In addition, several concepts and organizational aspects of Noxim have been reused. Examples are the Configuration Manager as well as the plug-in mechanism. In McAERsim, however, the latter enables the inclusion of different strategies for assigning neurons to computational nodes rather than the extension of available routing algorithms and selection strategies. In a similar vein, several components have been simplified, modified, or dropped to better match the address event representation (AER). At the same time, new features have been included, like support for multiple local processing elements, i.e., high-radix routers, as well as those that have already been discussed for the Noxim patch.

According to the router pipeline model of Section 2.3, transfer of AER packets is determined by the content of the shared connection memory, i.e., the TCAM and the SRAM modules. Prior to a simulation, their entries need to be populated. To simplify their initialization, a class called GlobalRoutingTable has been created. It reads a YAML file, with two nested dictionaries and lists as values at the lower level. Keys at the top level identify the node to which the entries at the lower level belong. Keys at the lower level correspond to source neuron IDs. The associated list finally identifies the outputs, to which the AER event has to be forwarded. During configuration, a router receives a pointer to the global routing table and extracts its local routing table based on its node ID. Then, by iterating over the entries of the local routing table, the TCAM and SRAM data structures get populated. The YAML file containing the global routing table is generated by an auxiliary binary called RTparser from NEST's connectivity file, which has been covered in Section 2.1. In fact, this binary has to perform two tasks in sequence to complete the conversion. First, it has to map neuron IDs to tile IDs. Based on this assignment and guided by a routing algorithm, it then needs to compute the required output activations at the source tile as well as all intermediate tiles, so that AER events from the source neuron can be transferred to all its target neurons. For these purposes, the binary additionally requires the file population_nodeids.dat from NEST's default outputs of the cortical microcircuit model as well as its own configuration file parser_config.yaml. Currently, only sequential mapping and dimension ordered XY routing are implemented. Options within parser_config.yaml are therefore limited to the number of neurons per processing element, the network dimensions, the number of processing elements per tile, the topology, and the desired output file name. Selectable topologies are TOPOLOGY_MESH and TOPOLOGY_TORUS.

McAERsim itself provides more flexibility with respect to neuron mapping and routing. Obviously, custom routing tables can be provided that follow a different (deterministic) routing strategy. In addition, there is the possibility to apply file based neuron assignment rather than sequential neuron mapping. The configuration file used in this case has a structure similar to the routing table. Keys at a top-level dictionary identify a processing element by its global ID. A key at an embedded dictionary then contains NEST's spike recorder file name that provides the AER events (source neuron IDs and time stamps). A processing element, however, usually only hosts a subset of these neurons. The list that is used as associated value at the second hierarchy level therefore contains the minimum and the maximum source neuron IDs taken into account. The choice of different neuron assignment strategies is enabled by the same plug-in mechanism that is used by Noxim for different routing and selection strategies, so that it can be easily extended. To pick one option, the global variables gnat_method and gnat_string have to be set accordingly, either via the configuration file config.yaml or by the respective command line arguments. The parameters determined this way, i.e., the spike recorder file names as well as the minimum and maximum source neuron IDs to be considered, take effect during the instantiation of the network initializing the local event queues of the processing elements. Such queues then contain AER events associated with the respective processing elements in chronological order and are queried by the canShot method to launch network traffic. It should be noted, however, that the AER events are still based on NEST's time stamps recorded in milliseconds. As explained in Section 2.2 for the Noxim patch, a conversion is needed for comparison with McAERsim's simulation time that is specified in picoseconds. The global variable nest_time_multiplier is defined for this purpose and can be varied to mimic an acceleration factor with respect to biological real time.

**Figure 7:**
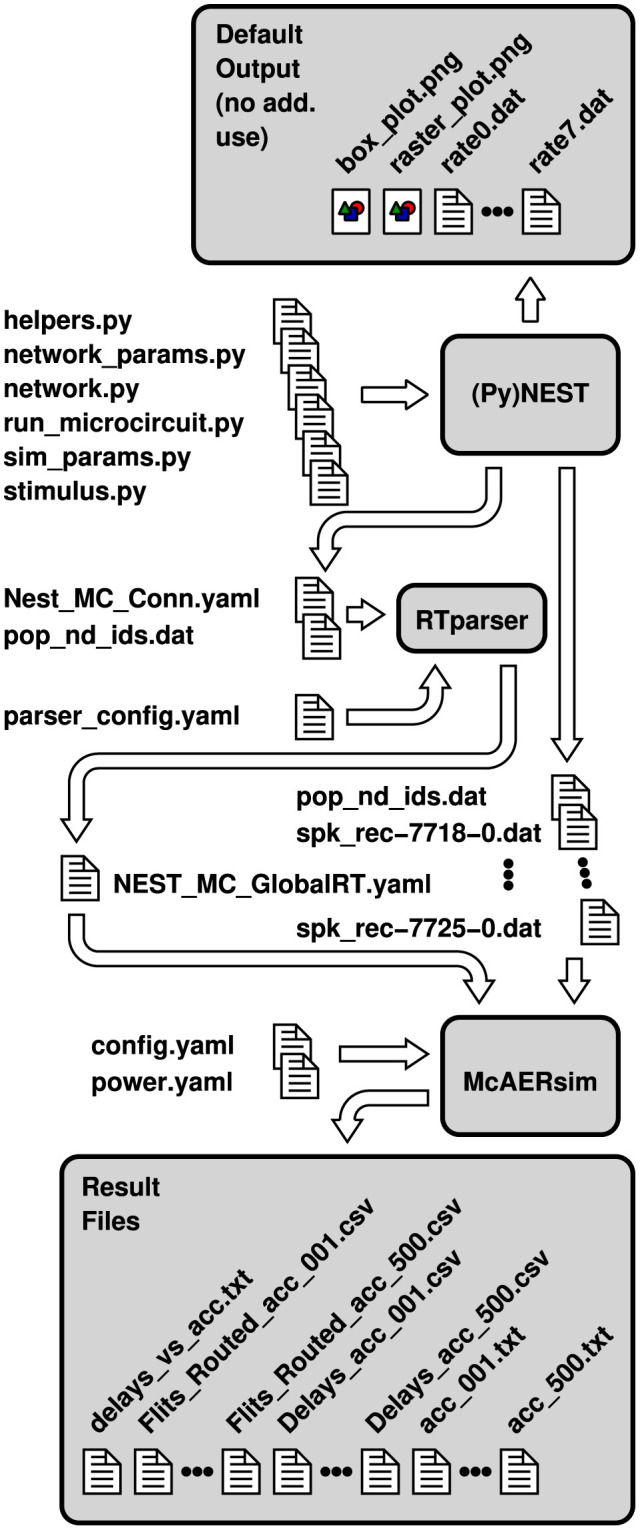
Configuration and result files involved in a co-simulation of NEST and McAERsim. File names are partially abbreviated—usually, they encode topology, size and the number of neurons per node as well.

Steps and data necessary for a co-simulation between NEST and McAERsim are summarized in Figure 7. As in Section 2.2, the workflow starts with slightly modified Python scripts originating from the pynest/examples/Potjans_2014 subdirectory of NEST. Executing run_microcircuit.py, PyNEST generates the default output files of the cortical microcircuit model as well as a file containing plain connectivity information (Nest_MC_Conn.yaml). In addition to the connectivity data, McAERsim and RTparser this time require some of the default output files, directly. Steered by parameters in its configuration file parser_config.yaml, RTparser composes a global routing table for McAERsim based on the connectivity information as well as the file population_nodeids.dat. The latter is required for the neuron to node mapping and has to be passed to McAERsim as well. Then, using the global routing table as well as AER events defined by NEST's spike recorder output files, a network simulation can be run by McAERsim to create the desired output statistics. As in Section 2.2, this simulation is controlled by parameters specified in the file config.yaml. Statistics on power consumption are already included in the command line outputs but currently default to zero. Once the contributions of different components have been assessed from RTL implementations or measurements, they may be entered into to the templates provided in power.yaml to yield realistic results.

Some care should be exercised to keep the parameters in parser_config.yaml and in config.yaml consistent. As for the co-simulation between NEST and Noxim in Section 2.2, a couple of parameters can not be selected freely but are predetermined or obey to some recommended values, e.g., to ensure comparability. This applies to the number of neurons per node, the network size, the buffer depth, and the address length of an AER event. Such events consist of single-flit packets, so that virtual channels have not been implemented, yet.

To step through different values of the global parameter nest_time_multiplier, a Bash script has been used that exploits the command line interface of McAERsim. Parsing overall statistics by sed, this yields the different result files shown in Figure 7.

## 3 Results

Data files obtained with the workflows presented in the previous section and by following the simulation setups outlined in subsequent subsections have been uploaded to Zenodo, see Robens (2024). In addition to the results from the network-on-chip simulations, also results from PyNEST simulations have been made available. For more details, please refer to the metadata of the data record.

### 3.1 Simulations with default settings

Development and initial simulation runs were conducted in a virtual machine running Ubuntu 20.04.2 LTS with 1.8GB of swap space and 4GB of assigned DRAM. For batch processing, i.e., to execute the Bash scripts examining network traffic caused by the cortical microcircuit scaled to 10%, the virtual machine was transferred to a server at which it was assigned 8GB of DRAM. As explained in the descriptions of the workflows that are illustrated in Figures 2, 7, first the output files of the PyNEST simulations were generated. Since default settings were applied, NEST run a pre-simulation covering 0.5s of biological real time. The actual simulation extended over 1s of biological real time and created the desired files. While stepping through the different values of the global variable nest_time_multiplier was controlled by the Bash scripts, several runs were initiated for different casting types and different topologies. Destination-address driven unicast (UC) and local multicast (LMC) were considered as well as source-address driven multicast (MC) and local multicast (LMC_SRC). All of them were applied to a mesh network and to a torus network. Most simulations followed the workflow of Figure 2, except for the simulations involving multicast routing that followed the workflow of Figure 7. Important parameter settings have already been discussed at the ends of Sections 2.2 and 2.4, while packet sizes were determined by the casting types as explained in Section 2.1. Due to the pre-simulation run by NEST, nevertheless it should be mentioned that time stamps within NEST's spike recorder files exhibit an offset of 500ms, whereas those in the YAML files used for data exchange between NEST and Noxim do not. McAERsim automatically does account for this offset, if the global variable nest_t_presim is set to the appropriate value—specified in milliseconds. Different simulation phases can be observed in Noxim and McAERsim as well. Conventionally, 1,000 reset cycles are executed first. Both simulators then suspend the computation of statistics for a number of cycles stored in the global variable stats_warm_up_time. Finally, the actual simulation is performed over a number of cycles as contained in the global variable simulation_time. Because of the simulation setup applied, there is no settling behavior, so that stats_warm_up_time was set to zero. To ensure that all packets can reach their destinations within the simulation time—at least in the case of a non-saturated network—, a guard interval was provided and simulation_time was set to 1.4 × 10^9^ cycles. However, for simulations emulating an acceleration factor, simulation_time was scaled in the same manner as the global variable nest_time_multiplier. The cycle time itself is specified in picoseconds and saved in the global variable clock_period_ps. It is set to 1,000 by default and was not altered for the simulations.^7^

**Figure 8:**
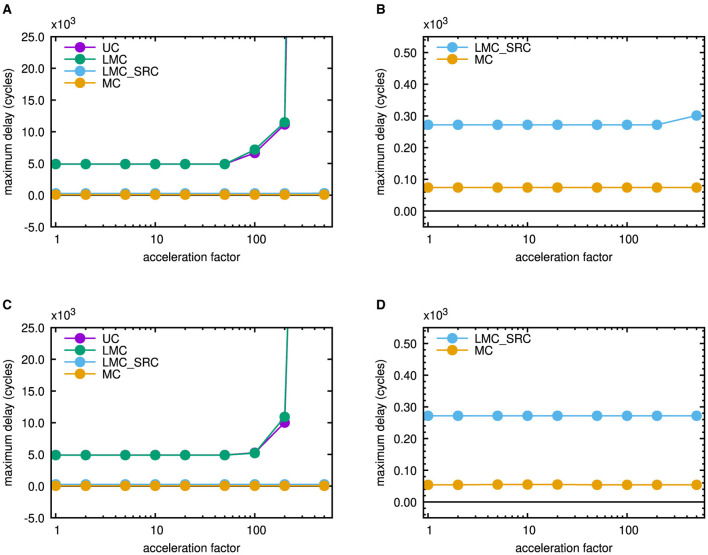
Maximum latency in a network simulation steered by traffic generated from the scaled microcircuit model of NEST (default scaling factor 10%) run over 1 s of biological real time. **(A)** 6 × 6 mesh network including all casting types. **(B)** Magnified view for the 6 × 6 mesh network including only source-address driven casting types. **(C)** 6 × 6 torus network including all casting types. **(D)** Magnified view for the 6 × 6 torus network including only source-address driven casting types.

During the execution of the Bash scripts, command line outputs of single simulation runs were redirected into text files termed acc_001.txt to acc_500.txt in Figures 2, 7. They contain aggregated statistics for the whole network and were parsed by the command line tool sed to extract the maximum delay for each acceleration factor. It should be mentioned that delay is the time interval between intended emission and reception of a header flit in Noxim. These information then were appended to a file referred to as delay_vs_acc.txt in the figures. By joint evaluation of individual delay_vs_acc.txt files for different casting types, Figures 8A, C were created. Figure 8A presents results for the mesh topology, while Figure 8C contains results for the torus topology.

They are comparable to “latency vs. injection load curves” that are conventionally applied to characterize network performance. The spike pattern, however, is application defined an does not change. Rather, the intervals between the spikes are scaled by the given factors to mimic an accelerated neuroscientific simulation. With respect to a fixed amount of cycles within the network-on-chip simulators, this can imply an increased traffic injection. As outlined in the introduction in relation to the Hybrid Neuromorphic Compute node of Trensch and Morrison (2022), the maximum admissible inter-node transmission latency is 500ns, if an acceleration factor of 100 is intended for the neuroscientific simulation. Despite the strong scaling applied to the neuroscientific test case, none of the destination-address driven casting types (UC or LMC) can meet this requirement, as can be seen from Figures 8A, C. Up to the onset of saturation at an emulated acceleration factor larger than 50 in both network topologies, the maximum latency for unicast is 4, 906ns, whereas it is 4, 898ns for local multicast. However, a comparison of Figures 8A, C shows that the torus topology can sustain its performance at higher emulated acceleration factors than the mesh topology. Both source-address driven casting types (LMC_SRC and MC), on the other hand, stay below the threshold of 500ns. Figures 8B, D therefore show magnified views of their behavior. Up to an acceleration factor of 200, the maximum latency in case of source-address driven local multicast is 272ns in both topologies. For larger acceleration factors up to 500, it stays at this value in case of the torus topology but raises slightly up to 301ns in case of the mesh topology. If multicast routing is applied, no saturation effects occur and the maximum latency stays at 74ns in case of the mesh topology, whereas it stays at an even lower value of 54ns in case of the torus topology. These plots are useful to monitor network saturation and to rule out casting schemes that can not meet a maximum target latency of 500ns.

**Figure 9:**
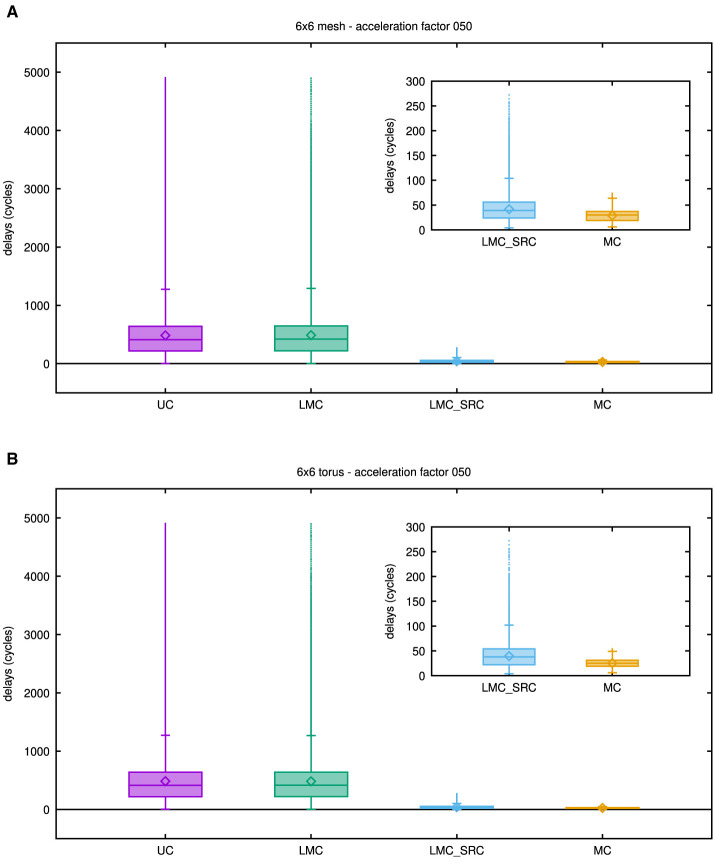
Boxplots showing latency results for different casting types: unicast (UC), destination-address driven local multicast (LMC), source-address driven local multicast (LMC_SRC), and multicast (MC). **(A)** Results for the 6 × 6 mesh network. **(B)** Results for the 6 × 6 torus network. To gain the results in these plots, an acceleration factor of 50 has been emulated, but as may be concluded from Figure 8, similar results can be obtained for smaller acceleration factors. The inset shows a magnified view of the results for the source-address driven casting schemes.

For acceleration factors below network saturation, it is also interesting to examine additional statistical key figures rather than just the maximum delay. Even though the latter is most critical in many cases, depending on the synchronization mechanism applied, overall simulation performance may depend on these attributes as well. Figures 9A, B therefore show boxplots of network latencies that were exported from the network-on-chip simulators for the mesh topology and the torus topology, respectively, while an acceleration factor of 50 was emulated.

In these plots, only outlier levels are depicted, while counts at the respective levels have no graphical representation. Insets zoom into the network latencies of source-address driven casting types, since they are much smaller than those of the destination-address driven casting types.

Although some differences are apparent, the destination-address driven casting types reveal rather similar statistical behavior with respect to their latencies. For these casting types, a large fraction of values is much smaller than the maximum delay value, as indicated by the individual third quartiles. Accordingly, the mean latency in these cases is almost an order of magnitude smaller than the maximum latency. By comparison to the maximum delay values stated in the previous paragraph, this can be verified with the aid of Table 1.

**Table 1:** Global average delay values using an emulated acceleration factor of 50.

	**Mesh**	**Torus**
UC	484.8ns	484.4ns
LMC	489.0ns	482.8ns
LMC_SRC	41.6ns	39.5ns
MC	27.8ns	24.5ns

While the median and mean latency values of the source-address driven casting types are not too far apart, the distributions of their latency values differ, which is especially true for the behavior of their outliers. As a consequence, the maximum delay value in the mesh topology is 3.7 times higher in case of source-address driven local multicast than in case of multicast, while this factor even raises to 5 in the torus topology. A final observation from Table 1 is that mean latency values for all casting types fall below the threshold of 500ns.

**Figure 10:**
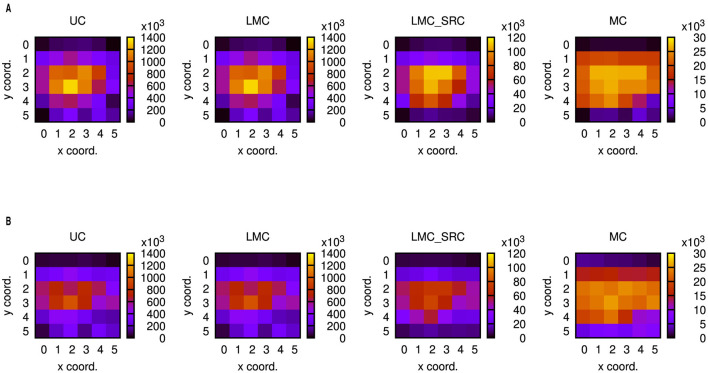
Number of routed flits for different casting types: unicast (UC), destination-address driven local multicast (LMC), source-address driven local multicast (LMC_SRC), and multicast (MC). **(A)** Results for the 6 × 6 mesh network. **(B)** Results for the 6 × 6 torus network. To gain the results in these plots, an acceleration factor of 50 has been emulated, but as may be concluded from Figure 8, similar results can be obtained for smaller acceleration factors.

Differences and similarities between the casting types, that have been discussed with respect to latencies, are also reflected by the spatial distribution of routing activity within the network in some manner. Accordingly, Figure 10 shows heatmaps of routed flits exported from the network-on-chip simulators for the different casting types and topologies observed at an emulated acceleration factor of 50.

It should be mentioned that routed flits in Noxim are only those forwarded to other network nodes and do not include the ones delivered to the local processing elements. McAERsim adapts to this definition but contains a comment in the source code enabling a simple change. At a first glance and in correspondence with the latencies, the number of routed flits is roughly an order of magnitude higher for the destination-address driven casting types than for the source-address driven casting types. Also, in all but the multicast cases, there are distinct hot spots close to the centers of the node arrangements. Consequently, few nodes carry a heavy load, while several nodes carry a light load, which parallels the observations regarding the latency variations. As opposed to that, network loading is much more uniform in the multicast cases. Comparing Figure 10A with Figure 10B, it becomes apparent that the number of routed flits in the hot spot areas is reduced by torus-shaped networks, while more transfers occur at the boundaries. Prevalently, this can be noticed to the west and to the east. Most likely, this is due to sequential mapping, which may cause populations to be split between nodes at opposite borders spaced one row apart. Communication is intense within a population, so that many packets are transferred through the center of the network in case of the mesh topology, while they take the wrap-around connections in case of the torus topology. In addition to the communication within a population, there is also communication between the populations. Because fewer packets are required in case of multicast routing, this may be the reason why an increased number of packets can also be observed at the south nodes of the torus topology in this case.

**Figure 11:**
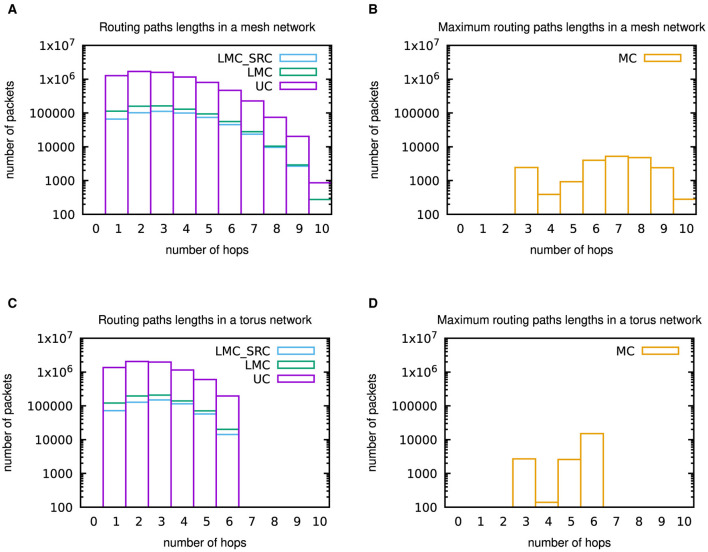
Histograms of (maximum) routing paths lengths within 6 × 6 mesh and torus networks. **(A)** Routing paths lengths in the mesh network for unicast (UC), destination-address driven local multicast (LMC), and source-address driven local multicast (LMC_SRC) routing. **(B)** Maximum routing paths lengths in the mesh network for multicast (MC) routing. **(C)** Routing paths lengths in the torus network for unicast (UC), destination-address driven local multicast (LMC), and source-address driven local multicast (LMC_SRC) routing. **(D)** Maximum routing paths lengths in the torus network for multicast (MC) routing. These results were not included in the outputs of the network-on-chip simulators. However, since deterministic XY routing has been applied during the network-on-chip simulations, they could be determined from the outputs of the NEST simulations by Python scripts.

Additionally, Figure 11 provides some insights into the routing paths lengths for the different casting types within the two topologies.

Those were not included in the outputs of the network-on-chip simulations, but thanks to the deterministic XY routing they can be calculated from the outputs of the NEST simulations available at Robens (2024) by Python scripts. For unicast (UC), destination-address driven local multicast (LMC), and source-address driven local multicast (LMC_SRC), all routing paths lengths information is included in the histograms, while only maximum paths lengths were considered in case of multicast (MC) routing. Results for the casting types in Figures 11A, C support the statement in the previous paragraph that communication is intense within a population, i.e., close to the source node. However, especially the maximum paths lengths results for multicast (MC) in Figures 11B, D underpin the verbatim citation from Heittmann et al. (2022) in Section 2.3 regarding the properties of the cortical microcircuit model, which implies that packets need to be transferred over the whole network in many cases. As has to be anticipated, a comparison of Figures 11A, B with Figures 11C, D reveals that the maximum number of hops is reduced by the torus topology.

The results on latency as well as the number of routed flits indicate the clear advantages that source-address driven multicast routing provides with respect to network traffic generated by NEST. In the following subsections, our contemplations are therefore limited to this casting type.

### 3.2 Simulations with multiple processing elements per tile

The excerpt of the YAML file used for data exchange between NEST and Noxim in Figure 1 points to a bursty nature of communication for the destination-address driven casting types, which was also observed from the simulations. Many packets get scheduled during a time step and need to be transferred through the network interface. Such queueing may add a significant contribution to the overall latency resulting in a potential bottleneck. The same issue with respect to the network interface controller (NIC) in case of unicast routing was also reported in (Jerger et al., 2008, p. 230). To improve this situation, a high-radix router connecting to a higher number of network interfaces may be applied. In a simple case, more and less powerful processing elements are used and associated with a tile, which host a smaller number of neurons. During the implementation of McAERsim, support for multiple processing elements per tile therefore has been taken into account.

**Figure 12:**
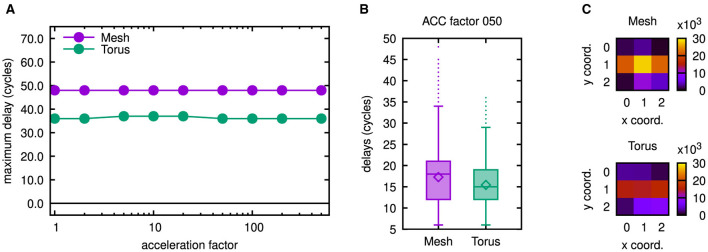
Latency results as well as number of routed flits for 3 × 3 mesh and torus networks. **(A)** Maximum latencies in the 3 × 3 networks. **(B)** Boxplots showing latency results for both 3 × 3 networks. **(C)** Number of routed flits in both 3 × 3 networks. To gain the results in **(B, C)**, an acceleration factor of 50 has been emulated, but as may be concluded from **(A)**, similar results can be obtained for smaller acceleration factors.

Figure 12 presents the results of a simulation with similar conditions like in the multicast cases of Figures 8–10, for which a sequential assignment of four processing elements per tile had been performed.

A row in the heatmaps of Figure 12 thus corresponds to two consecutive rows in the heatmaps of Figure 10. The diameter of the network is reduced this way, as are the maximum latencies shown in Figure 12A. From the boxplots of Figure 12B it may be concluded, that the same does apply to the delay distributions. In a physical realization, however, a larger switch will be required for a high-radix router. Thus, a higher latency of the switch traversal stage in the router pipeline needs to be expected. Accordingly, it may be necessary to increase the cycle duration. As a consequence, advantages gained by a higher number of processing elements per tile carefully need to be traded against the disadvantages caused by a higher router complexity.

### 3.3 Simulations with reduced scaling factor

**Figure 13:**
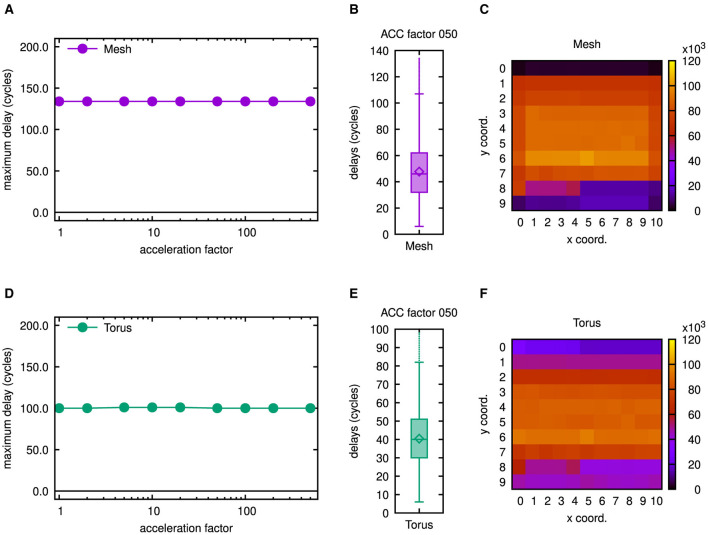
Latency results as well as number of routed flits for 11 × 10 mesh and torus networks. **(A)** Maximum latencies in the 11 × 10 mesh network. **(B)** Boxplot showing latency results for the 11 × 10 mesh network. **(C)** Number of routed flits in the 11 × 10 mesh network. **(D)** Maximum latencies in the 11 × 10 torus network. **(E)** Boxplot showing latency results for the 11 × 10 torus network. **(F)** Number of routed flits in the 11 × 10 torus network. To gain the results in **(B, C, E, F)**, an acceleration factor of 50 has been emulated, but as may be concluded from **(A, D)**, similar results can be obtained for smaller acceleration factors.

Experiments showed that co-simulations on the cortical microcircuit model can be run up to a scale of 17% with the server setup of the virtual machine as described at the beginning of Subsection 3.1. It was possible to extend the swap space of this virtual machine to 25GB and to assign 16GB of DRAM. Since memory had been increased roughly by a factor of 4 by these measures, we expected a co-simulation at a scale of 33% to be feasible. As confirmed by Figure 13, this simulation completed successfully.

Owing to the modified scaling, the number of neurons was increased by a factor of 3.3, so that the size of the network had to be increased approximately by a factor of 1.8 in each dimension. Accordingly, an arrangement of 11 × 10 tiles was used as can be seen from the heatmaps of Figures 13C, F. Because of the increased network dimensions, the maximum latency rose to 134 in case of the mesh topology and 100 or 101, respectively, in case of the torus topology. For different acceleration factors with respect to biological real time, this is shown in Figures 13A, D. Accordingly, the latency variation was increased by a factor roughly equal to the size of the network in one dimension as may be deduced from the interquartile ranges in Figures 13B, E, which were determined for an emulated acceleration factor of 50. Global average delay values went up slightly less than proportional and varied from 46.5 to 46.7 cycles in case of the mesh topology and from 39.5 to 39.7 cycles in case of the torus topology. Apart from the network dimensions, also the number of routed flits notably changed in the heatmaps of Figures 13C, F. However, the distribution of network load is still comparable to the multicast cases of Figure 10 and rather homogeneous as compared to the other casting types.

## 4 Discussion

In an attempt to reduce inter-node communication latencies by focusing on network traffic that is generated according to neuroscientific test cases directly, two simulation frameworks were proposed, in which network-on-chip simulators are interfaced with the neuroscientific development environment NEST. These setups were inspired by the design methodology SpiNeMap (Balaji et al., 2020), but beside of an interface to a different spiking neural network simulator, they add a couple of features to the constituent network-on-chip simulator Noxim. These features include gathering of further statistical data, data exports, as well as support for torus shaped networks and single-flit packets.

Presumably, these frameworks can be applied to biological network models up to the scale of the cortical microcircuit model, which sometimes is referred to as unit cell of the cortical network due to its distinct local structure. The frameworks may thus be considered a complement to the Python-based network simulator for large-scale heterogeneous neural networks presented in Kleijnen et al. (2022). For example, they may be applied to a distinct region of the multiarea model, that has been identified as potential bottleneck by the Python-based simulator, to provide more details on latencies—stimulating a hierarchical simulation approach. However, the cortical microcircuit itself may be considered challenging and consequently has been applied for benchmarking studies (van Albada et al., 2018). Therefore, it was selected as target application for the simulation experiments that have been conducted for different casting types and network topologies. The selection of a casting type is not supported in the released version of Noxim, but rather unicast routing is inherently assumed. However, fostered by the address event representation (AER), source-address driven and destination-address driven local multicast could be emulated by an appropriate packet organization. Multicast support in network-on-chip simulators, on the other hand, was argued to be scarce, at least for networks of sizes beyond those covered by routing masks. Therefore, a re-implementation of Noxim—called McAERsim—has been created around a source-address driven tree-based multicast router pipeline model for single-flit AER packets. In addition, support for multiple processing elements per tile was taken into account to address potential interface congestion.

The simulation experiments yielded three kinds of result display. In analogy to conventional “latency vs. injection load” curves, “maximum latency vs. acceleration factor” curves were created to assess network performance. The requirement of time stamp conversion between the simulators as well as the command line interfaces of the NoC simulators were exploited to emulate different acceleration factors with respect to biological real time and step through them by the aid of a Bash script. These plots could be used to rule out the destination-address driven casting types since their maximum latencies exceeded the desired threshold value of *T*_*COM*_ = 500ns almost by an order of magnitude even in a considerably scaled version of the cortical microcircuit and were affected by network saturation at higher acceleration factors. In addition, boxplots were generated, which provide more insights into the distributions of delays. Finally, heatmaps of routed flits were drawn, indicating the spatial distribution of network load. Both representations are in favor of multicast routing in a torus topology, because this combination leads to rather even distributions of network load and latencies, while it results in the smallest maximum delays.

The reduction of downscaling in the experiments by a factor of three caused the latencies to increase little more than the square root of this. If the same trend continues up to the full scale of the cortical microcircuit, the inter-node transmission latency stays well below a few hundred cycles. Under the premises of the simulations, thus, the threshold value of *T*_*COM*_ = 500ns would not be exceeded.

Using a higher-radix router to enable four processing elements per tile, the mesh size was reduced from 6 × 6 tiles to 3 × 3 tiles in a further experiment. However, since arbitration is performed between the inputs in McAERsim, the probability of collisions rises, which renders the size reduction less effective with respect to a latency improvement. Accordingly, the maximum delays were reduced by smaller factors than could be expected from the network size reduction. In addition, internal latencies of a higher-radix router tend to be larger, so that longer clock cycles might be required, which leads to a further penalty. As argued, in McAERsim support for multiple processor elements per tile is ineffective with respect to its initial purpose, because it does not alleviate interface congestion, but rather shifts it from the network interface to the interface of the connection memory. Yet, it might serve for this purpose in Noxim and can be exploited to reduce the network size. Furthermore, it allows for network configurations vaguely reminiscent of SpiNNaker's communication subsystem as detailed in Furber et al. (2006) and Plana et al. (2008)—lacking however several aspects like default and emergency re-routing.

**Figure 14:**
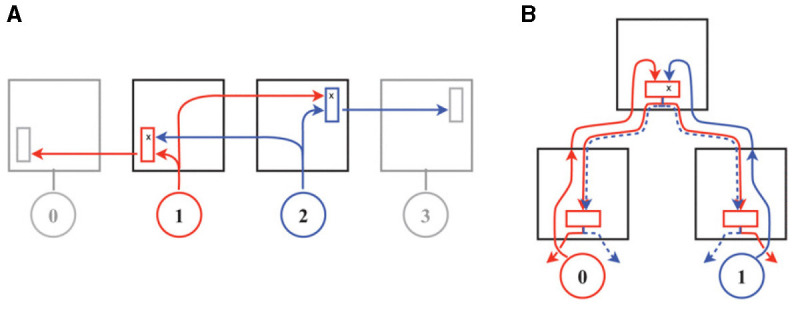
Switching-level deadlock in wormhole routing for multi-flit packets according to Merolla et al. (2014). In the mesh network of **(A)** both packets, ① and ②, acquire a grant in one direction but fail to acquire the grant in the other direction, so that progress stalls. This situation does not occur in the multicast binary-tree network of Neurogrid as shown in **(B)**, since arbitration precedes branching, when the latter is restricted to the downward phase. © 2014 IEEE. Reprinted, with permission, from Merolla, P., Arthur, J., Alvarez, R., Bussat, J.-M., and Boahen, K. “A Multicast Tree Router for Multichip Neuromorphic Systems,” IEEE Transactions on Circuits and Systems I: Regular Papers, Vol. 61, No. 3, pp. 820–833, Mar. 2014. doi: 10.1109/TCSI.2013.2284184.

As promoted by the address event representation (AER), McAERsim does rely on single-flit packets in order to convey information on spike events. With respect to its router implementation, this is essential in two ways: first, no special precautions for deadlock avoidance within the torus topology have been taken. Such had to be conceived for the Noxim patch, but in case of single-flit packets, channel allocations are released immediately after traversal by the header flit, so that no cyclic dependencies can arise. Second, and for the same reason, no measures to avoid switching-level deadlocks were devised. The latter can in principle occur in meshes, if multicast routing is applied, which was noted in Merolla et al. (2014) and is illustrated in Figure 14A (This situation is avoided by the sequence of actions in the multicast binary-tree network of Neurogrid as shown in Figure 14B.). According to Konstantinou et al. (2021), however, the forwarding of *single-flit packets* in each multicast router is—by construction—deadlock free. In this context, it is important to distinguish flits, used for flow control, and phits, i.e., the number of bits transferred in parallel within one cycle. As pointed out in the description of the example application in (Moradi et al., 2018, Section V), parallel input/output ports were realized through direct wire/pin connections on their board. In this case, both terms match. Usually, however, I/O pins are precious, so that the AER packets need to be (partially) serialized. According to Furber et al. (2006), SpiNNaker therefore uses 8-wire inter-chip links transferring 2-of-7 non-return-to-zero codes. Similarly, in Zamarreno-Ramos et al. (2013), an extension to their AER links via Rocket I/O serial interfaces with 8 b / 10 b encoding is presented that uses stop/resume messages for flow control. Since the number of phits is larger than the number of flits in these cases, either different clock speeds prior and after de-/serialization need to be used, or additional cycles need to be spend for these purposes. In the latter case, however, the duration of line traversal would be extended, which in turn increases the mandatory buffer depth, as was discussed in connection with the flow-control round-trip latency at the end of Section 2.2. Modeling this effect should be easily possible by making use of the Transaction Level Model (TLM) library of SystemC but is devoted to some future work. For the time being, parallel I/O as demonstrated by Moradi et al. (2018) will be assumed.

The proposed simulation frameworks, thus, are really useful to examine network performance based on application generated traffic. Neuroscientific test cases conveniently can be run in an embedded version of NEST to steer the simulations. At the same time, the constituent network-on-chip simulators feature plenty of options to modify important network parameters and observe the effects they have on characteristic key figures. To help direct the development of accelerator units intended for large-scale neuroscientific simulations, inter-node communication latency is of particular importance. The detailed analysis of a possible node-internal realization in Trensch and Morrison (2022) assessed that acceleration factors with respect to biological real time in the order of 10–50 may be possible, while it sets a limit of *T*_*COM*_ = 500ns on the inter-node communication latency for this purpose. Observing the results on maximum latencies that we obtained from the cortical microcircuit model at different scaling factors and extrapolating from them, these acceleration factors, indeed, seem to be within reach. So far, however, these results are biased by some assumptions that are inherent to the network-on-chip simulators, parallel inter-node links being the most striking one. Therefore, it will be interesting to experiment with the clock speed and to address the additional delay imposed by de-/serialization in a small extension to McAERsim in some future work.

## Data availability statement

The datasets presented in this study can be found in online repositories. The names of the repository/repositories and accession number(s) can be found in the article/supplementary material.

## Author contributions

MR: Writing—original draft, Software, Investigation, Conceptualization. RK: Writing—review & editing. MS: Writing—review & editing, Funding acquisition. SW: Writing—review & editing.

## Footnotes

^1^In addition, it gathers further statistical data, features some data exports, and adds support for torus shaped networks (currently only with XY routing).

^2^Source codes and simulation scripts used in this work are available online at: https://github.com/mrobens/nenocsi-mcaersim/tree/nenocsi and https://github.com/mrobens/nenocsi-mcaersim/tree/mcaersim. Please also check the Supplementary material.

^3^A traffic queue is a C++ std::queue with entries of type NestTraceComm. This is a struct that contains relevant parameters of a spike communication, i.e., source and destination node, the number of flits sent and in total as well as the NEST time stamp converted to simulation cycles.

^4^Note that the sequence of commands in the source code of Noxim agrees more to the output-first virtual channel allocator of (Dimitrakopoulos et al., 2013, Figure 18B) than to the input-first virtual channel allocator of (Dimitrakopoulos et al., 2013, Figure 18A), which may intuitively be associated with Figure 3B.

^5^For the ease of implementation, a C++ std::set with Integer data type is used instead of a bit-vector to represent the digital word, while a C++ std::vector of this data type represents the whole SRAM in McAERsim.

^6^With regard to the amount of required resources, this may be necessary in order to prepare for more advanced routing algorithms or emergency re-routing, as discussed in Lester and Richards (2008). For dimension ordered XY routing, source addresses delivered to the inputs of a router are contained in disjoint sets, so that a shared connection memory may not be mandatory. Then, however, arbitration would be shifted to a later stage, where performance may be traded against implementation complexity.

^7^This corresponds to a router running at 1GHz, which seems reasonable with respect to an application specific implementation. To examine a router design designated for an FPGA, the clock frequency should be reduced, i.e., clock_period_ps should be increased.
