# Supplementary material for: NoC simulation steered by NEST: McAERsim and a Noxim patch
Source: Front Neurosci. 2024 Jun 20;18:1371103. doi: 10.3389/fnins.2024.1371103 (PMC11222605; doi:10.3389/fnins.2024.1371103)
Supplement: Supplementary file 1 [file Data_Sheet_1.PDF]

Supplementary Material

Source codes and simulation scripts used in the main article are available online at: https://github.com/mrobens/nenocsi-mcaersim/tree/nenocsi and https://github.com/mrobens/nenocsi-mcaersim/tree/mcaersim. For the creation of these releases, modifications to the files helpers.py as explained in section 2.1 of the main article and referred to later on have been separated out into auxiliary Python scripts helpers_aux.py. The installation instructions as well as the installation scripts contained in these releases do already account for this change.

SIMULATIONS WITH DEFAULT SETTINGS

By following the instructions of the INSTALL.txt files in the doc subfolders of these releases or by using the installation scripts ubuntu-20.04.2-install.sh contained in their scripts/install subfolders, everything will be set up in such a way, that execution of the ”Bash scripts” sweep_acc_factor_<topology>_<casting_type>.sh will create the data reported in section ”3.1 Simulations with Default Settings” of the main article. Note that <topology> and <casting_type> are placeholders for which the respective topologies - mesh or torus - and the respective casting types - UC, LMC, LMC_SRC or MC - have to be substituted. Look into the bin/results/data_<topology>_<casting_type> subfolders of the installation directories to locate the data.

SIMULATIONS WITH REDUCED SCALING FACTOR

In section ”3.3 Simulations with Reduced Scaling Factor” of the main article, only results generated by McAERsim have been considered. Prior to the steps described below, you may want to create a copy of the respective configuration files, so that you can always revert your changes. To enable simulations with a reduced downscaling factor, first the Python scripts of NEST’s cortical microcircuit model need to be adapted. Change to the nest_inputs subfolder of McAERsim’s installation directory and edit the file network_params.py. In the net_dict dictionary, change the value associated with the key ’N_scaling’ from 0.1 to 0.33. Similarly, change the value associated with the key ’K_scaling’ from 0.1 to 0.33. Then, edit the file sim_params.py. In the sim_dict dictionary, change the value associated with the key ’print_time’ from True to False. If not done before - by following the installation instructions or by executing the installation script -, load the environment variables required by NEST. To do so, change to the bin subdirectory of NEST’s installation directory and issue the command source nest_vars.sh. Change back to the nest_inputs subfolder of McAERsim’s installation directory. At this point, if it is already present in this folder, you may want to rename the data subfolder. To run the cortical microcircuit model type python3 run_microcircuit.py. Now, change to the bin subfolder of McAERsim’s installation directory. Edit the file parser_config.yaml. Change the value associated with the key mesh_dim_x from 6 to 11. Similarly, change the value associated with the key mesh_dim_y from 6 to 10. Generate the new global routing table by executing the command ./rtparser ../nest_inputs/data/population_nodeids.dat ../nest_inputs/data/NEST_MC_Connections.yaml. Edit the file parser_config.yaml again, toggle the value associated with the key topology from TOPOLOGY_TORUS to TOPOLOGY_MESH, or vice versa, and update the output file name associated with the key

output_file_name accordingly. Then, execute rtparser again using the same command as above. Next, edit the file config.yaml. Inspect the string value associated with the key gnat_string. By default, this string ends with spike_recorder-7718-0.dat. Change this part to spike_recorder-25465-0.dat while keeping the rest unmodified. At this point, if it is already present, you may want to rename the subfolder results to keep the data of previous simulation runs. Finally, change to the scripts subfolder of McAERsim's installation directory. Edit the simulation script sweep_acc_factor_mesh_MC.sh and change the variable definition DIM="6x6" to DIM="11x10". Do the same with the simulation script sweep_acc_factor_torus_MC.sh. Then, execute both scripts to create the simulation results at the reduced downscaling factor.

SIMULATIONS WITH MULTIPLE PROCESSING ELEMENTS PER TILE

Also section "3.2 Simulations with Multiple Processing Elements per Tile" of the main article focuses on output generated by McAERsim. In order to re-create this output, it is most easiest to start with the default settings. This time, the same results of NEST can be used, as have been generated using the default settings. To put them back in place, change to the subfolder nest_inputs of McAERsim's installation directory. If you followed the instructions of the previous section, a new data subfolder will be present, which you may want to rename. The data subfolder gained from the simulation run using default settings should have been preserved under a modified name. Then, you can copy this folder and name it data again. Enter the bin subfolder of McAERsim's installation directory and edit the file parser_config.yaml. Change the value associated with the key mesh_dim_x from 6 to 3. Change the value associated with the key mesh_dim_y from 6 to 3 as well. In addition, increase the value associated with the key number_pes from 1 to 4. Then execute the command ./rtparser ../nest_inputs/data/population_nodeids.dat ../nest_inputs/data/NEST_MC_Connections.yaml. Edit the file parser_config.yaml again, toggle the value associated with the key topology from TOPOLOGY_TORUS to TOPOLOGY_MESH, or vice versa, and update the output file name associated with the key output_file_name accordingly. Then, execute rtparser again using the same command as above. These two steps should overwrite the two files NEST_MC_Global_RT_Mesh.yaml and NEST_MC_Global_RT_Torus.yaml in the nest_inputs/data subdirectory of McAERsim's installation directory, so that they contain global routing tables suitable for the modified network topology. Next edit the Makefile in the bin directory. Uncomment the definition #SPECIAL := -DNO_PES = 2 and change the respective value from 2 to 4. Now, you need to re-build McAERsim. To do so, type make clean, make, and make rtparser. After this re-build, edit the file config.yaml and change the values associated with the keys proc_arr_dim_x and proc_arr_dim_y from 1 to 2. At this point, if it is present, you may want to rename the subfolder results to keep previous simulation results. Change to the scripts subfolder of McAERsim's installation directory. Then, edit the simulation script sweep_acc_factor_mesh_MC.sh and update the variable definition DIM="6x6" to DIM="3x3". Do the same with the simulation script sweep_acc_factor_torus_MC.sh. Finally, execute both scripts to create the simulation results taking into account four processing elements per tile.
